# Aseptic Meningitis and Its Viral Etiologies, Clinical Characteristics and Management Practices in Children: A Retrospective Hospital-Based Study From Jordan

**DOI:** 10.7759/cureus.24383

**Published:** 2022-04-22

**Authors:** Amira Masri, Arwa Dwaikat, Nour Haroun, Lubna Haikal, Malik Kharabsheh, Amira Daher, Faris Bakri, Abdelkarim Al Qudah

**Affiliations:** 1 Pediatrics, The University of Jordan, Amman, JOR; 2 Medicine, The University of Jordan, Amman, JOR; 3 Medicine/Infectious Disease, The University of Jordan, Amman, JOR

**Keywords:** viral panel, polymerase chain reaction, jordan, cerebrospinal fluid, enterovirus

## Abstract

Purpose

In this study, we aimed to describe the clinical characteristics, laboratory findings, aetiologies, and role of PCR in the decision on the management plan and duration of hospital stay in Jordanian children diagnosed with aseptic meningitis.

Methods

This retrospective observational cohort study included children diagnosed with meningitis who were admitted to the paediatric ward at Jordan University Hospital (JUH) during the period from January 2016 to August 2020. Patients were identified through the ICD9 discharge code of meningitis. Patients diagnosed with aseptic meningitis (defined as a patient with signs and symptoms of meningitis with a cerebrospinal fluid (CSF) white cell count of >5 cells/mm^3^, and a negative CSF Gram stain) were included, while patients who had low CSF glucose (<50% of serum) positive cerebrospinal fluid Gram stain and/or culture for bacterial meningitis were excluded.

Files were reviewed to collect data on the clinical picture, viruses identified by the CSF viral polymerase chain reaction (PCR) panel, duration of medication, and hospital stay in patients with identified virus versus those with negative viral PCR.

Results

One hundred and thirty-one patients were included: 87 males (66.4%) and 44 females (33.5%). Fever was the most common presenting symptom, followed by headache, vomiting, and excessive sleep in 48.0%, 42.7%, and 35.8% of the patients, respectively. Prior oral antibiotic use was reported in 48/125 (38.4%) patients.

White blood cell count (WBC) ranged from 4.800 to 22.000. cells/mL, 45 patients (34.3%) had counts above 15.000 cells/mL. C-reactive protein level was high in 61/103 (59.2%) patients. CSF WBC count was <100 in 62 (47.3%) patients while neutrophils predominance of >70% was present in 27 (20.6%) patients. Viral panel PCR was done for 100/131 (76.3%) patients and was positive in 66/100 (66%) patients; with enterovirus being the most common identified viruses (60/100; 60%).

The average duration of hospital stay was 5.9 and 5.5 days for those with negative and positive PCR respectively. Ten (7.6%) patients had seizures upon presentation. None of the patients had any neurological sequel related to his meningitis.

Conclusion

Enteroviruses are the most common identified cause of paediatric aseptic meningitis in Jordan. Although PCR revealed an identified virus in around half of the patients, nevertheless, there was no adjustment in the management plan regarding duration of empirical antibiotic use and hospital stay. Increasing knowledge and awareness among clinicians on viral meningitis’ lab characteristics might have great impact on duration of hospital stay and thus would be reflected on the patient and the healthcare system as well.

## Introduction

Meningitis is a major cause of mortality and morbidity in children and can be related to a bacterial pathogen or to aseptic meningitis; a term used to describe meningitis that is caused by aetiologies other than bacteria [1], with viral aetiologies being the most common [2]. Although bacterial meningitis is more dangerous than viral meningitis; however, viral meningitis may also lead to neurological sequels [3] and is more common than bacterial meningitis, especially after the vaccine era [4]. Furthermore, while most viral meningitis carries a benign course, hospitalisation is often prolonged because of the unclarity of the responsible aetiology.

Aetiologies of meningitis may differ according to geographic location and endemicity of pathogens [3,5,6]. Several studies on the aetiologies and the clinical characteristics of viral meningitis in children have been reported from different countries, with limited data available from developing/low-resource countries including Jordan [7]. Polymerase chain reaction (PCR) testing is specific and can help differentiate viral from bacterial meningitis and thus might help adjust the management plan [8,9]. However, in developing and low-resource countries, the impact of the utility of routine use of viral PCR while investigating children diagnosed with meningitis and its role in modifying the management plan was not explored before.

In this study, we aimed to describe the clinical characteristics, laboratory findings, aetiologies, and role of PCR in the decision on the management plan and duration of hospital stay in Jordanian children diagnosed with aseptic meningitis.

## Materials and methods

This is a retrospective observational cohort study that included children who were diagnosed to have meningitis and were admitted to the paediatric ward at Jordan University Hospital (JUH) during the period from January 2016 to August 2020. Patients were identified through the ICD9 discharge code of meningitis. Patients diagnosed with aseptic meningitis (defined as a patient with signs and symptoms of meningitis with a CSF white cell count of >5 cells/mm3, and a negative CSF Gram stain [1]) were included while patients who had low CSF glucose (< 50% of serum) and or positive cerebrospinal fluid gram stain and or culture for bacterial meningitis were excluded.

Files were reviewed to collect data on the age and sex of patients, presenting a clinical picture, viruses identified based on the CSF viral polymerase chain reaction (PCR) panel, duration of medication, and hospital stay in patients with identified virus versus those with negative viral PCR.

Statistics

Data were analysed using Microsoft Excel 2016. Continuous data were summarised as average, median values, and categorical data as n (%).

Ethical approval

This study was approved by the Institutional Review Board (IRB) of JUH (1/2016).

## Results

We identified 131 patients with aseptic meningitis, ten of whom did not fully meet the definition of aseptic meningitis (as they had CSF WBC count < 5 cells/ mm^3^) but were included because they had signs and symptoms of meningitis including fever and positive meningeal signs and were managed as meningitis; 4/10 had enterovirus as revealed by PCR. There were 87 males (66.4%) and 44 females (33.5%) with a male to female ratio of 1.9:1.

The age of patients ranged from one month to 14 years; 23 (17.2%) were in the first year of life, 51 (38.9 %) were between >1 and <6 years, 47 (35.0%) were between ≥6 and 12 years, and 10 (7.6%) were >12 years of age.

Clinical manifestations 

Fever (ranging from 38 to 40 degrees Celsius) was the most constant presenting symptom in all the patients, followed by headache, vomiting, and excessive sleep in 48.0%, 42.7%, and 35.8% of the patients, respectively. Table 1 summarises all the associated clinical manifestations.

**Table 1:** Clinical characteristics of the 131 patients

Clinical presentation	N of patients (%)
Fever (documented or undocumented )	131(100%)
Headache	63 ( 48.0%)
Vomiting	56 (42.7% )
Excessive sleep	47 (35.8 %)
Poor appetite	34 (25.9%)
Flue like symptoms	33 (25.1%)
Photophobia	22 (16.7%)
Irritability	14 ( 10.6%)
Papilledema	
Present	7/76 (9.2%)
Ophthalmic consult not done	55/131 (41.9%)
Nausea	11 (8.3%)
Diarrhoea	7 (5.3%)
Rash	7 (5.3%) two of them had chicken pox and one had oral herpetic lesions
Change in level of consciousness	4 (3.0%)
Seizures	10 ( 7.6%)
Use of antibiotics prior to presentation to hospital	
Yes	48/125 (38.4%)
Data missing from file	6/131 (4.5%)

Flu-like symptoms were reported in 33 (25.1%) patients, oral antibiotic use prior to diagnosis of meningitis was reported in 48/125 (38.4%) patients (data was missing from the files of 6/131 patients).

Laboratory investigations 

Blood Tests

White blood cell (WBC) count ranged from 4.800 cells/mL to 22.000 cells/mL. Around half of the patients (65; 49.6%) had WBC counts below 10.000 cells/mL while around one third (45; 34.3%) had counts above 15,000 cells/mL.

Blood sodium upon presentation: was normal in most patients, hyponatremia (sodium level ranging from 131-134 mEq/L was present in 26 (19.8%) while only one patient had hypernatremia (sodium level of 164 mEq/L). Spot sodium in the urine was missing from the files of most patients.

C-reactive protein (CRP) level of CRP ranged from normal (<5 mg/L) to 101 mg/L and was high (>5 mg/L) in 61/103; 59.2% of patients, levels above 20 mg/L were reported in around one-third of the patients (35/103; 33.9%).

Cerebrospinal Fluid (CSF)

Around half of the patients (62; 47.3%) had CSF WBC count of <100, levels >1,000 were present in two patients (1.5%) and were mainly lymphocyte (90%).

While CSF percentage of lymphocytes ranged from 0% to 100%, neutrophil predominance of >70% was present in 27 (20.6%) patients. All patients had normal CSF glucose levels.

Viral panel PCR (panel included enterovirus, herpes simplex virus 1 and 2, varicella zoster, parechovirus, and mumps virus) was done for 100/131 (76.3%) patients and was positive in 66/100 (66%) patients; enterovirus being the most identified viruses (60/100; 60%). Table 3 shows the details of the CSF results.

**Table 2:** CSF results of the 131 patients

CSF variable	N (%)	Comments
WBC count		
< 5	10 (7.6%)	
6-100	62 (47.3%)	
101-500	47 (35.8%)	
501-1000	9 (6.8%)	
>1000	2 (1.5%)	
Missing data from file	1 (0.76%)	
Viral panel results		
Viral panel not done	23/131 (24.4%)	
Viral panel done	100/131 (76.3%)	
Negative Viral panel	34/100 (34%)	
Positive viral panel	66/100 (66%)	
Enterovirus	60/100 (60%)	
Varicella zoster	3/100 (3.0%)	
Equivocal varicella zoster	1/100 (1.0%)	
Equivocal mumps	2/100 (2.0%)	

Neuroimaging Studies 

Brain CT scan was done for 22 (15.2%) patients, while brain MRI was done for 12 (9.1%); four patients had abnormal brain MRI findings (white matter changes ongoing with acute disseminated encephalomyelitis in two, previously known basal ganglia signal abnormality in a patient previously suspected to have mitochondrial disorder, a previously known patient with hydrocephalous and ventriculoperitoneal shunt).

**Table 3:** Lab Investigations and Neuroimaging

Investigation	N (%)
Serum white blood cells /ml( WBC)	
< 10.000	65 (49.6%)
10.000-15.000	21(16.0%
> 15.000	45 (34.3%)
Blood sodium	
Normal	105 (80.1%)
Low (130-134 )	26 (19.8%)
High (>145)	1 (0.76%)
C reactive protein (CRP)	
Done	103/131 (78.6%)
Normal (<5)	41/103 (39.8%)
High >5	61/103 (59.2%)
6-20	27/103 (26.2%)
>20	35/103 (33.9%)

Treatment, complications, and outcome 

 All patients had received empirical antibiotic treatment for meningitis (as this is the routine in our hospital for meningitis; third-generation cephalosporin and vancomycin), 10/131 (7.6%) received in addition empirical antiviral treatment (acyclovir). Two patients also received methylprednisolone as neuroimaging showed evidence of acute disseminated encephalomyelitis. The average duration of empirical antibiotic treatment was 5.9 days for those with negative PCR result and 5.5 days for those with identified virus by PCR.

Ten (7.6%) patients had seizures upon presentation ongoing with a probable concurrent encephalitis, three were known to have a previous neurological disorder but they did not have a previous history of epilepsy; one of them was discharged on antiepileptic medication due to his underlying previously known neurological condition. None of the patients had any neurological sequel related to his meningitis.

## Discussion

In this study, we have identified 131 patients diagnosed with aseptic meningitis. Aseptic meningitis is currently the most common cause of paediatric meningitis [10-12], as vaccines have contributed significantly to the drop in childhood bacterial meningitis in the world [4].

We noticed that males were more commonly to be affected than females. The mechanism underlying this male predominance which was also noticed by other previously reported studies is unknown [13-15]. However, the X chromosome expression could be responsible for the immunological protection of females due to the X-linked microRNAs-related processes and could partly explain the increased risk of infections in males [16].

While fever was dominant, the clinical presentations and associated symptoms varied in our patients. Headache and vomiting were present in only half of the patients, and around one-quarter of patients had flu-like symptoms. Our findings are ongoing with previous reports in the literature as children were found to present with non-specific febrile illness and were less likely to present with meningeal complaints than adults [15,17] rendering the diagnosis of aseptic meningitis in children a real challenge. Furthermore, 38.4% of patients in our study received oral antibiotics prior to presentation complicating further the diagnostic challenge. Antibiotic use prior to lumbar puncture in children with aseptic meningitis is not an uncommon practice and was reported in the literature in around one-third of paediatric patients [15]. This common use of prior antibiotic therapy is probably related to the overlapping clinical picture with upper respiratory tract infections and the common lack of typical symptoms of meningitis in children [15].

In addition, initial blood and CSF tests including WBC and CRP were often high confirming that it is often difficult initially to differentiate between bacterial and aseptic meningitis based on initial blood and CSF tests [18]. Our finding that 20.6% of the patients had CSF neutrophilic predominance is ongoing with previous studies which revealed that viral meningitis can present with neutrophilic pleocytosis in 25% of patients [15]. Furthermore, Jaijakul et al. found that 47% of patients with enteroviral meningitis had neutrophilic pleocytosis [19].

Aseptic meningitis can be due to infectious or non-infectious aetiologies, viruses being the most common identified aetiologies in the literature [17]. An identified viral aetiology was present in 66% of our patients. Although much progress has been made in diagnostic methods, nevertheless an identified viral aetiology for aseptic meningitis can be found in only 30% to 65% of patients [20,21].

Enterovirus was the most common identified virus causing aseptic meningitis in our study ongoing with previous studies [22]. Mixed infections are rare, thus the identification of viral pathogen by PCR should mandate discontinuation of antibiotics and discharge of the patient [23]. Although the routine use of a viral panel in cases with suspected meningitis may help decrease the total length of hospital stay [24], nevertheless, in our study there was no significant difference in the average length of hospital stay between those patients with an identified viral aetiology and those without an identified aetiology. This current practice could be related to either a delay in obtaining the PCR result or mistrust of clinicians, especially in patients who had a high CSF white blood cell count or neutrophilic predominance. Increasing knowledge and awareness, shortening the time of laboratory PCR results, and expanding the viral panels may help improve current practices.

Although fundus examination was a routine practice prior to LP, neuroimaging was only ordered based on clinical indications, which were often related to an abnormal neurological exam or seizures. Previous studies found that although neuroimaging is commonly used in aseptic meningitis; however, it is commonly normal and does not alter management [25]. A recent study by Salazar et al. in 2017 documented the lack of adherence of clinicians to the Infectious Diseases Society of America guidelines and confirms the lack of utility of obtaining cranial imaging in the absence of specific indications [25].

All patients in our study were discharged without any neurological sequel related to aseptic meningitis. The acute seizure was a very rare presentation in our patients, however, children with viral meningitis presenting with acute seizures have an increased later risk to develop unprovoked seizures and epilepsy [26]. Furthermore, some studies have shown evidence that brain infections in childhood may compromise early brain development and might increase the risk of epilepsy and other neurodevelopmental disorders later in life thus these patients might need further future to follow up [27-30].

## Conclusions

This study has several limitations being retrospective, hospital-based and involving only one tertiary referral hospital in Amman. Nevertheless, this study revealed several important points. First, enteroviruses are the most common identified cause of paediatric aseptic meningitis in Jordan. Secondly, although PCR revealed an identified virus in around half of the patients, nevertheless, there was no adjustment in the management plan regarding the duration of empirical antibiotic use and hospital stay. Increasing knowledge and awareness among clinicians on viral meningitis’ lab characteristics might have a great impact on the duration of hospital stay and thus would be reflected on the patient and the healthcare system as well.

## References

[1] Tapiainen T, Prevots R, Izurieta HS (2007). Aseptic meningitis: case definition and guidelines for collection, analysis and presentation of immunization safety data. Vaccine.

[2] Logan SA, MacMahon E (2008). Viral meningitis. BMJ.

[3] Carod-Artal FJ, Wichmann O, Farrar J, Gascón J (2013). Neurological complications of dengue virus infection. Lancet Neurol.

[4] McIntyre PB, O’Brien KL, Greenwood B, Van De Beek D (2012). Effect of vaccines on bacterial meningitis worldwide. Lancet (London, England).

[5] Koyuncu OO, Hogue IB, Enquist LW (2013). Virus infections in the nervous system. Cell Host Microbe.

[6] Soares CN, Cabral-Castro MJ, Peralta JM, de Freitas MR, Zalis M, Puccioni-Sohler M (2011). Review of the etiologies of viral meningitis and encephalitis in a dengue endemic region. J Neurol Sci.

[7] Gaudenzi G, Kumbakumba E, Rasti R (2020). Point-of-care approaches for meningitis diagnosis in a low-resource setting (Southwestern Uganda): Observational cohort study protocol of the “PI-POC” trial. JMIR Res Protoc.

[8] (2022). Case definitions for infectious conditions under public health surveillance. https://pubmed.ncbi.nlm.nih.gov/9148133/.

[9] Kadambari S, Okike I, Ribeiro S, Ramsay ME, Heath PT, Sharland M, Ladhani SN (2014). Seven-fold increase in viral meningo-encephalitis reports in England and Wales during 2004-2013. J Infect.

[10] Boudet A, Pantel A, Carles MJ (2019). A review of a 13-month period of FilmArray meningitis/encephalitis panel implementation as a first-line diagnosis tool at a university hospital. PLoS One.

[11] Radmard S, Reid S, Ciryam P (2019). Clinical utilization of the FilmArray meningitis/encephalitis (me) multiplex polymerase chain reaction (PCR) assay. Front Neurol.

[12] Naccache SN, Lustestica M, Fahit M, Mestas J, Dien Bard J (2018). One year in the life of a rapid syndromic panel for meningitis/encephalitis: a pediatric tertiary care facility’s experience. J Clin Microbiol.

[13] Ben Abid F, Abukhattab M, Ghazouani H (2018). Epidemiology and clinical outcomes of viral central nervous system infections. Int J Infect Dis.

[14] Aldriweesh MA, Shafaay EA, Alwatban SM, Alkethami OM, Aljuraisi FN, Bosaeed M, Alharbi NK (2020). Viruses causing aseptic meningitis: a tertiary medical center experience with a multiplex PCR assay. Front Neurol.

[15] Shukla B, Aguilera EA, Salazar L, Wootton SH, Kaewpoowat Q, Hasbun R (2017). Aseptic meningitis in adults and children: diagnostic and management challenges. J Clin Virol.

[16] Pinheiro I, Dejager L, Libert C (2011). X-chromosome-located microRNAs in immunity: might they explain male/female differences? The X chromosome-genomic context may affect X-located miRNAs and downstream signaling, thereby contributing to the enhanced immune response of females. Bioessays.

[17] Hasbun R (2000). The acute aseptic meningitis syndrome. Curr Infect Dis Rep.

[18] Ray P, Badarou-Acossi G, Viallon A, Boutoille D, Arthaud M, Trystram D, Riou B (2007). Accuracy of the cerebrospinal fluid results to differentiate bacterial from non bacterial meningitis, in case of negative gram-stained smear. Am J Emerg Med.

[19] Jaijakul S, Salazar L, Wootton SH, Aguilera E, Hasbun R (2017). The clinical significance of neutrophilic pleocytosis in cerebrospinal fluid in patients with viral central nervous system infections. Int J Infect Dis.

[20] Kupila L, Vuorinen T, Vainionpää R, Hukkanen V, Marttila RJ, Kotilainen P (2006). Etiology of aseptic meningitis and encephalitis in an adult population. Neurology.

[21] Frantzidou F, Kamaria F, Dumaidi K, Skoura L, Antoniadis A, Papa A (2008). Aseptic meningitis and encephalitis because of herpesviruses and enteroviruses in an immunocompetent adult population. Eur J Neurol.

[22] Rotbart HA (2000). Viral meningitis. Semin Neurol.

[23] Marshall GS, Hauck MA, Buck G, Rabalais GP (1997). Potential cost savings through rapid diagnosis of enteroviral meningitis. Pediatr Infect Dis J.

[24] Wootton SH, Aguilera E, Salazar L, Hemmert AC, Hasbun R (2016). Enhancing pathogen identification in patients with meningitis and a negative Gram stain using the BioFire FilmArray(®) meningitis/encephalitis panel. Ann Clin Microbiol Antimicrob.

[25] Salazar L, Hasbun R (2017). Cranial imaging before lumbar puncture in adults with community-acquired meningitis: clinical utility and adherence to the Infectious Diseases Society of America guidelines. Clin Infect Dis.

[26] Misra UK, Tan CT, Kalita J (2008). Viral encephalitis and epilepsy. Epilepsia.

[27] Muhle RA, Reed HE, Stratigos KA, Veenstra-VanderWeele J (2018). The emerging clinical neuroscience of autism spectrum disorder: a review. JAMA Psychiatry.

[28] Mazina V, Gerdts J, Trinh S (2015). Epigenetics of autism-related impairment: copy number variation and maternal infection. J Dev Behav Pediatr.

[29] Singh G, Prabhakar S (2008). The association between central nervous system (CNS) infections and epilepsy: epidemiological approaches and microbiological and epileptological perspectives. Epilepsia.

[30] Gau SS, Chang LY, Huang LM, Fan TY, Wu YY, Lin TY (2008). Attention-deficit/hyperactivity-related symptoms among children with enterovirus 71 infection of the central nervous system. Pediatrics.

